# Supplementary material for: West Nile Virus in the State of Ceará, Northeast Brazil
Source: Microorganisms. 2021 Aug 10;9(8):1699. doi: 10.3390/microorganisms9081699 (PMC8401605; doi:10.3390/microorganisms9081699)
Supplement: Supplementary file 1 [file microorganisms-09-01699-s001.zip › Table S2.pdf]

Table S2. Amplification of WNV-seropositive free-ranging bird species collected at PLI, Boa Viagem, CE.

Species	#Specimens Captured	C	#Specimens Tested	S	Prop. (95% CI)	A
Forpus xanthopterygius	2	0.7%	2	50%	0.50 (0.09-0.91)	1,750
Turdus rufiventris	4	1.5%	4	25%	0.25 (0.05-0.70)	937.5
Thraupis sayaca	20	7.6%	19	10.5%	0.10 (0.03-0.30)	838
Columbina talpacoti	19	7.2%	11	9.1%	0.09 (0.02-0.38)	596.2

A (amplification capacity) = C (Relative abundance) × S (seroprevalence)^{2}.
