# Supplementary material for: West Nile Virus in the State of Ceará, Northeast Brazil
Source: Microorganisms. 2021 Aug 10;9(8):1699. doi: 10.3390/microorganisms9081699 (PMC8401605; doi:10.3390/microorganisms9081699)
Supplement: Supplementary file 1 [file microorganisms-09-01699-s001.zip › Table S3.pdf]

Table S3. Amplification of WNV-seropositive free-ranging bird species collected at Farm 1, Boa Viagem, CE.

Species	#Specimens Captured	C	#Specimens Tested	S	Prop. (95% CI)	A
Thlypopsis sordida	2	1.7%	1	100%	1.00 (0.21-1.00)	17,000
Columbina talpacoti	2	1.7%	2	50%	0.50 (0.09-0.91)	4,250
Turdus rufiventris	2	1.7%	2	50%	0.50 (0.09-0.91)	4,250
Furnarius figulus	4	3.5%	4	25%	0.25 (0.05-0.70)	2,187.5
Passer domesticus	68	59.1%	62	4.8%	0.05 (0.02-0.13)	1,361.7
Coryphospingus pileatus	9	7.8%	8	12.5%	0.12 (0.02-0.47)	1,218.7

A (amplification capacity) = C (Relative abundance) × S (seroprevalence)^{2}.
