# Supplementary material for: West Nile Virus in the State of Ceará, Northeast Brazil
Source: Microorganisms. 2021 Aug 10;9(8):1699. doi: 10.3390/microorganisms9081699 (PMC8401605; doi:10.3390/microorganisms9081699)
Supplement: Supplementary file 1 [file microorganisms-09-01699-s001.zip › Table S4.pdf]

Table S4. Mosquito species captured at distinct environments and with different methods in Boa Viagem, Ceará, Brazil, in September 2019.

A: Sum of mosquitoes caught in periurban and rural areas of Boa Viagem, Ceará

Mosquito species	Inside houses			Close to houses									Open fields									
				Chicken sheds			Horse barns						Human attraction					CDC				
	Asp.		**Total**	CDC		Total	Asp.		CDC		Total	**Total**	Directly with nets		Shannon trap		Total			Total	**Total**	**Grand Total**
	♀	♂		♀	♂		♀	♂	♀	♂			♀	♂	♀	♂		♀	♂			
Ad. squamipennis				1	1	2			1	1	2	**4**						4		4	**4**	**8**
Ae. aegypti	7	7	**14**																		**0**	**14**
Ae. fluviatilis													1				1				**1**	**1**
Ae. scapularis													2				2				**2**	**2**
An. albitarsis s.l.	1	1	**2**															12	7	19	**19**	**21**
An. oswaldoi s.l.																			1	1	**1**	**1**
An. triannulatus s.l.				1		1			1		1	**2**						9	0	9	**9**	**11**
Anopheles (*Nys.*) sp.	1		**1**												1		1	12		12	**13**	**14**
Cq. nigricans	1		**1**	1		1						**1**				1	1	8		8	**9**	**11**
Cq. veneuelensis	1		**1**																		**0**	**1**
Cx. amazonensis																		3		3	**3**	**3**
Cx. chidesteri																		5	7	12	**12**	**12**
Cx. coronator Complex									4		4	**4**	6				6	5		5	**11**	**15**
Cx. declarator	5		**5**	1		1			2		2	**3**	1				1	29	2	31	**32**	**40**
Cx. nigripalpus				1		1			2	1	3	**4**	1	1			2	14		14	**16**	**20**
Cx. quinquefasciatus	72	52	**124**	2	3	5	1	1	3	9	14	**19**	4	1			5	7	2	9	**14**	**157**
Cx. (*Cux.*) sp.				4		4			1		1	**5**						19		19	19	**24**
Cx. inhibitator																14	14				**14**	**14**
Cx. panacossa										1	1	**1**	1				1	4	12	16	**17**	**18**
Cx. ribeirensis (?)																		4		4	**4**	**4**
Culex (*Mel.*) sp.	1		**1**										9		6		15	36	4	40	**55**	**56**
Culex spp.				2		2			2		2	**4**	4				4	11	2	13	**17**	**21**
Ma. indubitans	7		**7**	3		3			1		1	**4**	25		1		26	33	1	34	**60**	**71**
Ma. pseudotitillans	1		**1**	1		1						**1**	21				21				**21**	**23**
Ma. titillans	6	4	**10**	26	39	65	3	1	9	5	18	**83**	94	1	18		113	27	3	30	**143**	**236**
Mansonia sp.	7	1	**8**	13	2	15				5	5	**20**	9	4			13	6	5	11	**24**	**52**
Ur. lowii																		1		1	**1**	**1**
Uranotaenia sp.				1		1			1		1	**2**									**0**	**2**
Total	110	65	**175**	57	45	102	4	2	27	22	55	**157**	178	7	26	15	226	249	46	295	**521**	**853**

Asp.: backpack aspirator; CDC: CDC light trap.

B: Mosquitoes captured in the periurban areas corresponding to the probable local of infection of the WNV index case and surroundings at Boa Viagem, Ceará.

Mosquito species	Inside houses Asp. ♀	Inside houses Asp. ♂	Inside houses Total	Close to houses: Chicken sheds CDC ♀	Chicken sheds CDC ♂	Chicken sheds Total	Horse barns Asp. ♀	Horse barns Asp. ♂	Horse barns CDC ♀	Horse barns CDC ♂	Horse barns Total	Close to houses Total	Open fields: Human attraction Directly with nets ♀	Directly with nets ♂	Shannon Trap ♀	Shannon Trap ♂	Human attraction Total	CDC ♀	CDC ♂	CDC Total	Open fields Total	**Grand Total**
Ad. squamipennis				1	1	2			1	1	2	**4**						1		1	**1**	**5**
Ae. aegypti	7	7	**14**																		**0**	**14**
Ae. fluviatilis													1				1				**1**	**1**
Ae. scapularis													2				2				**2**	**2**
An. oswaldoi s.l.																			1		**1**	**1**
An. triannulatus s.l.				1		1			1		1	**2**						2	0	2	**2**	**4**
Anopheles (*Nys.*) sp.	1		**1**												1		1				**1**	**2**
Cq. nigricans				1		1						**1**				1	1				**1**	**2**
Cx. amazonensis																		3		3	**3**	**3**
Cx. chidesteri																		5	7	12	**12**	**12**
Cx. coronator Complex									4		4	**4**	6				6	5		5	**11**	**15**
Cx. declarator	3		**3**	1		1			2		2	**3**	1				1	28	2	30	**31**	**37**
Cx. nigripalpus				1		1			2	1	3	**4**	1	1			2	13		13	**15**	**19**
Cx. quinquefasciatus	70	52	**122**	2	3	5	1	1	3	9	14	**19**	4	1			5	4	1	5	**10**	**151**
Cx. (*Cux.*) sp.				4		4			1		1	**5**						19		19	**19**	**24**
Cx. inhibitator																14	14				**14**	**14**
Cx. panacossa										1	1	**1**	1				1	4	12	16	**17**	**18**
Cx. ribeirensis (?)																		4		4	**4**	**4**
Cx. (*Mel.*) sp.	1		1										9		6		15	36	4	40	**55**	**56**
Culex spp.				2		2			2		2	**4**	4				4	9	2	11	**15**	**19**
Ma. indubitans				3		3			1		1	**4**	25		1		26	1		1	**27**	**31**
Ma. pseudotitillans	1		**1**	1		1						**1**	21				21				**21**	**23**
Ma. titillans	2	1	**3**	26	39	65	3	1	9	5	18	**83**	94	1	18		113	21	3	24	**137**	**223**
Mansonia sp.		1	**1**	13	2	15				5	5	**20**	9	4			13	2	1	3	**16**	**37**
Uranotaenia sp.				1		1			1		1	**2**									**0**	**2**
	85	61	**146**	57	45	102	4	2	27	22	55	**157**	178	7	26	15	226	157	33	189	**416**	**719**

Asp.: backpack aspirator; CDC: CDC light trap.

C: Mosquitoes captured in the rural area, namely Farm1, located around 5km from the probable local of infection of the WNV index case in Boa Viagem, Ceará.

Mosquito species	Inside houses Asp. ♀	Inside houses Asp. ♂	**Total**	Open fields CDC ♀	Open fields CDC ♂	**Total**	**Grand total**
Ad. squamipennis				3		**3**	**3**
Anopheles (*Nys.*) sp.				12		**12**	**12**
An. albitarsis s.l.	1	1	**2**	12	7	**19**	**21**
An. triannulatus s.l.				7		**7**	**7**
Cq. nigricans	1		**1**	8		**8**	**9**

Cq. veneuelensis	1		**1**				**1**
Cx. nigripalpus				1		**1**	**1**
Cx. quinquefasciatus	2		**2**	3	1	**4**	**6**
Cx. declarator Complex	2		**2**	1		**1**	**3**
Cx. spp.				2		**2**	**2**
Ma. indubitans	7		**7**	32	1	**33**	**40**
Ma. titillans	4	3	**7**	6		**6**	**13**
Mansonia sp.	7		**7**	4	4	**8**	**15**
Ur. lowii				1		**1**	**1**
**TOTAL**	25	4	**29**	92	13	**105**	**134**

Asp.: backpack aspirator; CDC: CDC light trap.
