# Supplementary figures and images for: West Nile Virus in the State of Ceará, Northeast Brazil
Source: Microorganisms. 2021 Aug 10;9(8):1699. doi: 10.3390/microorganisms9081699 (PMC8401605; doi:10.3390/microorganisms9081699)

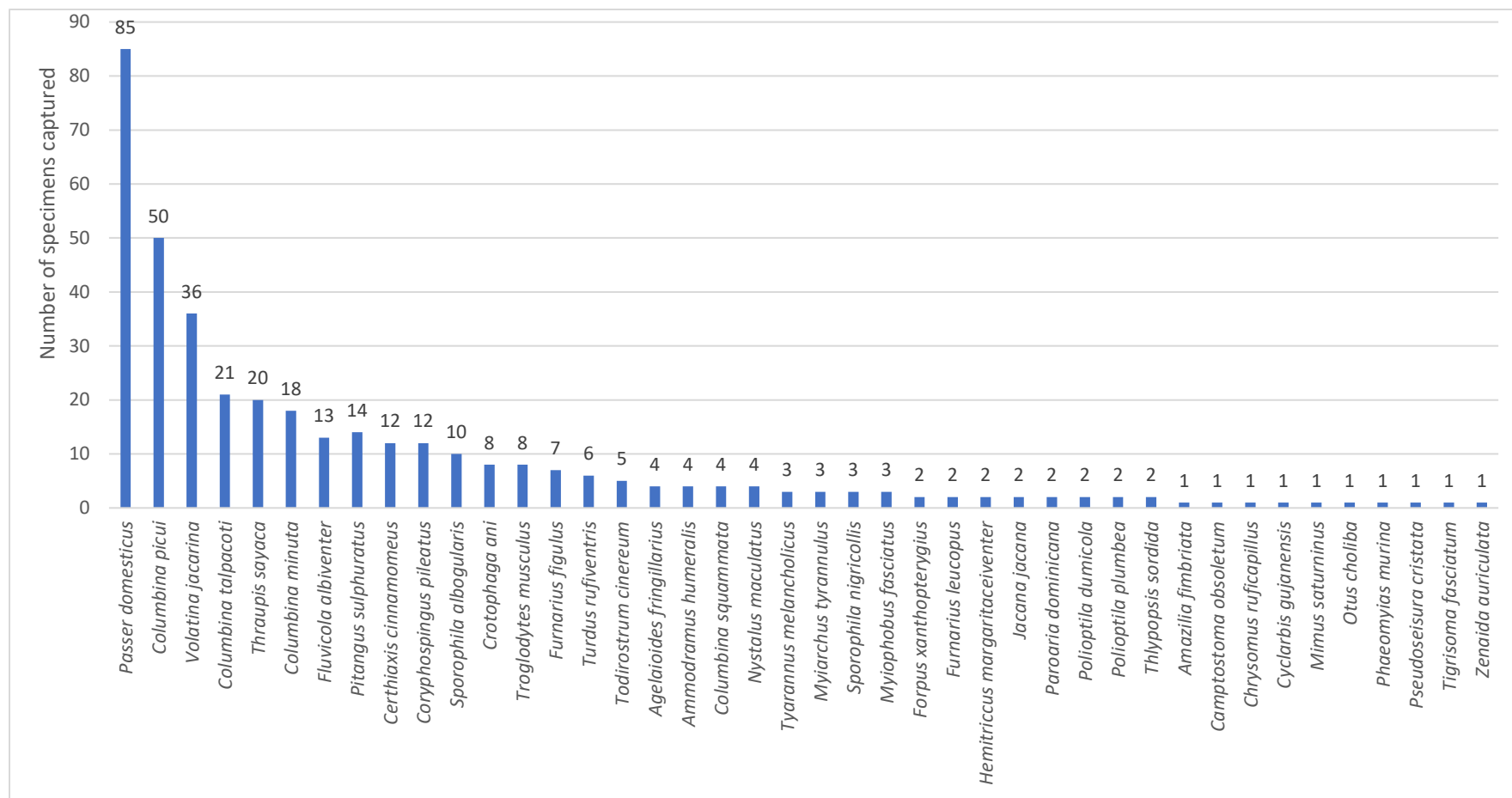

**Figure S1.** Specimens of free-ranging wild birds captured in Boa Viagem, CE in September 2019.

Supplement: Supplementary file 1 [file microorganisms-09-01699-s001.zip › Figure S1.pdf]
